# Association between cadmium and anti-Mullerian hormone in premenopausal women at particular ages

**DOI:** 10.1186/s40557-018-0255-7

**Published:** 2018-07-09

**Authors:** Yu min Lee, Hye Won Chung, Kyungah Jeong, Yeon-Ah Sung, Hyejin Lee, Shinhee Ye, Eun-Hee Ha

**Affiliations:** 1 Department of Occupational and Environmental Medicine, Ewha Womans University School of Medicine, 1071, Anyangcheon-ro, Yangcheon-gu, Seoul, 07985 Republic of Korea; 2 Department of Obstetrics and Gynecology, School of Medicine, Ewha Womans University, Seoul, South Korea; 3 Department of Internal Medicine, School of Medicine, Ewha Womans University, Seoul, South Korea

**Keywords:** Anti-Mullerian hormone, Cadmium, Premenopausal women, Ages

## Abstract

**Background:**

Anti-Mullerian hormone (AMH) in women is secreted by granulosa cells of antral follicles. AMH appears to be a very stable marker for ovarian function. It may be used to diagnosis cases of premature ovarian failure, polycystic ovary syndrome (PCOS), and ovarian tumors. It has been suggested that cadmium exposure can reduce female fecundity. The purpose of this study was to investigate whether environmental exposure to cadmium was associated with alterations in AMH with regards to age.

**Methods:**

In a cross-sectional study, the data of premenopausal women living in Seoul, ranging from 30 to 45 of age was collected. The study included a total of 283 women who completed serum AMH and whole blood cadmium assessments. Linear regression analyses were used in order to examine the association between cadmium and AMH. Given that age was the strongest confounder in both cadmium and AMH concentrations, we stratified subjects by 5 years old and analyzed their data.

**Results:**

Geometric mean concentrations of blood cadmium and AMH were 0.97 μg/L and 3.02 ng/ml, respectively. Total association between cadmium and AMH was statistically significant (adjusted coefficient = − 0.34 (0.15), *p* = 0.02). After stratification, the only age group with a negative association between cadmium and AMH were the women raging between 30 and 35 years (adjusted coefficient = − 0.43 (0.18), *p* = 0.01).

**Conclusions:**

The results of this study suggest that environmental exposure to cadmium may alter the AMH level of premenopausal women, depending on their age group.

## Background

Anti-Müllerian hormone (AMH) in women is secreted by granulosa cells of antral follicles [1, 2]. AMH is a member of transforming growth factor-beta (TGF-β) superfamily. It appears to be a very stable marker for ovarian function. It might be useful for diagnosing cases of premature ovarian failure, polycystic ovary syndrome (PCOS), and ovarian tumors [1, 3, 4]. It has been reported that the number of primordial follicles is related to their activation rate. This is reflected by the number of growing follicles. This type of relationship might be useful as an indicator of ovarian reserve. Cadmium is known to be an endocrine disruptor. It has been suggested that exposure to cadmium reduces fecundity [5–7]. The reproductive toxicity of cadmium is mediated through various pathways, such as structural damage to testicular vascular structure and blood-testis barrier, cytotoxicity to Sertoli and Leydig cells, oxidative stress, apoptosis, and disruption of the hypothalamus-pituitary gonadal axis in males [8]. However, recent studies have solely reported on the effects of cadmium exposure with regards to the reproductive capacity of males. Few studies have reported the health effects of cadmium has on women. In addition, studies have shown that the exposure to cadmium in humans increases with age, while the concentration of AMH in women decreases with age. Therefore, the purpose of this study was to investigate whether cadmium exposure was related to changes in AMH in premenopausal women according to age.

## Methods

### Study participants

We conducted a cross-sectional survey of premenopausal women in Seoul, Republic of Korea. The women volunteered after seeing ads on bulletin boards in various places, including the Ewha Womans University Medical Center, Healthy Families Support Center, Community Health Center, Community Service Center, and Community Blog for Mothers. In order to investigate the effects of environmental exposure on premenopausal women’s health, the 308 participants, ranging in age from 30 to 49 years old, were recruited from September to November 2014. To exclude women who might have already undergone menopause, subjects 45 years of age and older were excluded (*n* = 13) [9]. It has been determined that PCOS (polycystic ovarian syndrome) patients are associated with high AMH levels [2]. Therefore, PCOS patients were excluded from the study (n = 1). In the process of measuring AMH and cadmium, it was not possible measure below or above the limit of detection (LOD) in some cases. These cases were also excluded (*n* = 11). Therefore, a total of 283 women were included in this study.

Data concerning covariates was extracted from questionnaires, physical examinations and pelvic ultrasonography by trained researchers and nurses. We examined the following potential confounders: premenopausal women’s age (30–35, 35–40, 40–45 years), body mass index (BMI), parity (nulliparous, 1, 2, 3, 4), smoking status (current smoker, past smoker, never smoker), and fish intake (less than half a fish, half a fish, or more than one fish per one meal). This study protocol was approved by the Institutional Review Board of the Ewha Womans University Medical Center. Written informed consent was obtained from each participant.

### Cadmium assessment

Venous blood was collected from premenopausal women using EDTA treated tubes. The blood samples were frozen and stored at 4–8 °C throughout the entire analysis period. All analyses were conducted within three days of storage at the Seoul Medical Science Institute.

Analysis of the cadmium content in blood was performed using a Zeeman Atomic Absorption Spectrometer (AAS 280Z, Agilent, USA). The Spectrometer was verified using certified standard and quality control materials. In short, 100 μl of 1% nitric acid and 1.5 ml of diluent were added to 100 μl of the blood sample and vortexed. In order to prepare the diluent, Triton X-100 5 ml, 20% NH4H2PO4 10 ml, and HNO3 1 ml were added to 1 l of water (HPLC grade). The detection limit of cadmium (three times the standard deviation of the SD; SD * 3) was 0.015 μg/L, and the limit of cadmium quantification (10 times standard deviation of the SD; SD * 10) was 0.05 μg/L. ClinChek Whole Blood Controls level I and level II (RECIPE, GERMANY) were measured before, during, and after the analysis. The value of the Coefficient of Variation(CV) (%) of the quality control substances for level I and level II were precisely 6.1 and 5.7%, respectively. The analysis accuracy of the blood cadmium analysis was verified by the cadmium survey (five times a year) conducted by the College of American Pathologists(CAP) and Lead and Multielement Proficiency(LAMP) Survey (three times a year) by the Centers for Disease Control and Prevention(CDC).

### AMH assessment

The concentrations of AMH were assessed with V MAX (microplate reader, Molecular Devices, USA). It was verified using certified standard and quality control materials. The detection limit of AMH (SD * 3) ranged from 0.08 ng/mL to 21.00 ng/mL. Its limit of detection (SD * 10) was 0.17 μg/L. AMH Gen II enzyme-linked immunosorbent assay (Beckman Coulter, USA) was used for the AMH measurement before, during, and after the assay. The CV (%) values of the level control materials were precisely 7.7 and 5.8% for level I and level II, respectively.

### Statistical analysis

Descriptive statistics for the participants characteristics were expressed as the number (%) or geometrical mean and standard deviation. Geometric means for cadmium and AMH concentrations were calculated and analyzed using the Wilcoxon rank sum test or Kruskal–Wallis rank sum test in order to determine the significant difference between the following covariate groups. After descriptive analyses, cadmium and AMH concentrations were transformed logarithmically due to their skewed distribution.

We used regression analysis in order to examine the effect of cadmium levels on the AMH level in premenopausal women within each age group. In an effort to select covariates for inclusion in multivariate models, we performed a literature review in order to identify the risk factors associated with cadmium exposure or AMH. The following key covariates were used in this study: Body Mass Index (BMI), parity, smoking status, and fish intake. It has been reported that the level of AMH varies according to age, and the level of AMH is changing in diseases such as PCOS and early ovarian failure [1]. Thus, AMH levels have been reported to be significantly lower in obese women (high BMI) [10, 11]. Smoking is a major source of cadmium exposure [12]. Cadmium exposure occurs mainly through diet, such as grains, fish, and more. In addition, heavy metals such as cadmium are increased in aquatic environments through industrial processes and fertilizers and can be introduced to humans through fish [13]. Moreover, fish is also a source of other compounds, such as vitamin D, which is associated with serum AMH [14]. Some participants had missing data for a given variable. They were excluded from the multivariate analysis. In order to examine the interactive effects of the age of premenopausal women and cadmium levels on AMH, we included an interaction term between cadmium level and age in the multivariate analysis. After identifying the interaction effects, stratified analysis was performed for each age group (in five years interval). All statistical analyses were carried out using SAS statistical software version 9.4 (SAS Institute Inc., Cary, NC, USA).

## Results

Participant characteristics were described in Table 1.

**Table 1:** Characteristics of participants

	N(%) or mean ± standard deviation	*p*-value*
Total	283	
Age (year)	36.22 ± 3.86	<.0001
30 < ≤35	135 (47.70)	
35 < ≤40	108 (38.16)	
40 < ≤45	40 (14.13)	
Missing	0	
BMI (kg/m2)	22.26 ± 3.12	
Missing	1	
Parity (NO.)		<.0001
0	9 (3.19)	
1	94 (33.33)	
2	150 (53.19)	
3	27 (9.57)	
4	2 (0.71)	
Missing	1	
Smoking status		<.0001
Current smoker	5 (1.79)	
Past smoker	27 (9.64)	
Never smoker	248 (88.57)	
Missing	3	
Fish intake per serving		<.0001
Less than half a fish	146 (52.33)	
Half a fish	99 (35.48)	
More than one fish	34 (12.19)	
Missing	4	

Data are presented as number (%), mean ± SD

*BMI* body mass index

*P-value for Chi square

We identified 283 individuals who were 30–45 years old after excluding those who were diagnosed with PCOS and those whose cadmium and AMH levels were beyond LOD. After descriptive analyses, cadmium and AMH concentrations were then logarithmically transformed because of their skewed distribution. Geometric mean levels of cadmium and AMH concentrations were 0.97 μg/L and 3.02 ng/ml, respectively (Table 2).

**Table 2:** Distribution of cadmium and anti mullerian hormone

	N(%)	Cadmium (ug/L)				Anti-Müllerian hormone (ng/ml)			
		Median [1Q, 3Q]	GM	GSD	P-value	Median [1Q, 3Q]	GM	GSD	*P*-value
Total	283	0.96 [0.77, 1.22]	0.97	1.44		3.62 [1.88, 6.01]	3.02	2.61	
Age (year)					<.0001				<.0001
30 < ≤35	135 (47.70)	0.86 [0.70, 1.12]	0.88	1.44		4.64 [2.58, 6.75]	4.19	2.07	
35 < ≤40	108 (38.16)	1.00 [0.82, 1.25]	1.02	1.40		3.41 [1.86, 5.39]	3.01	2.27	
40 < ≤45	40 (14.13)	1.16 [0.88, 1.47]	1.15	1.46		1.17 [0.31, 2.54]	1.00	3.20	

Data are presented as number (%), Median [1Q, 3Q]

*GM* geometric mean, *GSD* Geometric standard deviation. 1Q = 1st quartile. 3Q = 3^rd^quartile

Tested by p-value calculated by the Wilcoxon rank sum test or Kruskal–Wallis rank sum test because of the skewness of cadmium and anti-mullerian hormone

**Fig. 1:**
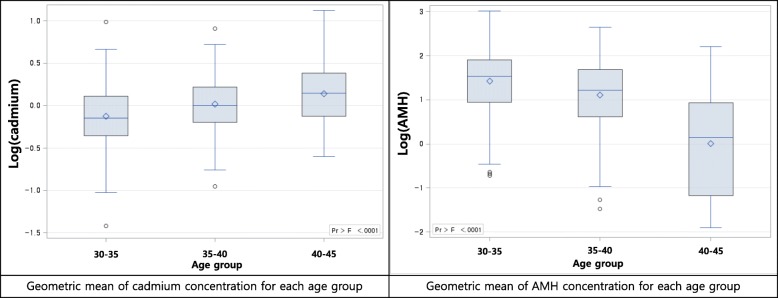
Distribution of cadmium and anti-mullerian hormone concentration with subgroup. AMH: Anti-Mullerian hormone

The differences in cadmium and AMH concentrations between age groups were calculated using the Wilcoxon rank sum test or Kruskal–Wallis rank sum test (Fig. 1).

**Fig. 2:**
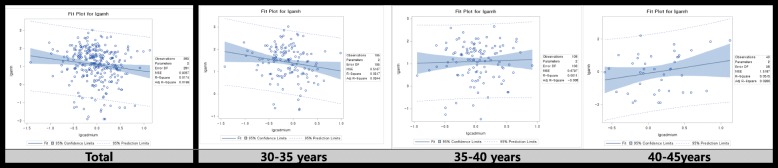
Graphs of simple regression serum cadmium concentration and anti-mullerian hormone concentration with subgroup

We then tested whether or not cadmium level was associated with the AMH level by using adjusted linear regression models (Fig. 2 and Table 3). Cadmium exposure decreased AMH level in the total group (β = − 0.34193 (0.14990), *p*-value = 0.0233). After stratification of the patients at 5-year intervals, the cadmium level decreased AMH levels in the age group of 30–35-year age group (β = − 0.43462 (0.17599), *p*-value = 0.0149).

**Table 3:** Linear regression with serum cadmium concentration and anti-mullerian hormone concentration

	Anti mullerian Hormone									
	N	Crude	95% CI		*P*-value	N	Adjusted^a^	95% CI		*P*-value
Total	283	−0.34385 (0.15428)	−0.64754	− 0.04015	0.0266	276	−0.34193 (0.14990)	− 0.63706	− 0.04679	0.0233
BY age										
30 < ≤35	135	−0.35569 (0.17052)	−0.69296	− 0.01841	0.0389	131	−0.43462 (0.17599)	− 0.78298	−0.08625	0.0149
35 < ≤40	108	0.08280 (0.23812)	−0.38929	0.55488	0.7287	106	0.14879 (0.23103)	−0.30968	0.60726	0.5211
40 < ≤45	40	0.69231 (0.48192)	−0.28328	1.66791	0.1590	39	0.94249 (0.48586)	−0.04717	1.93216	0.0613

^a^Adjusted for BMI, parity, smoking status, and fish intake

## Discussion

In this study, we determined the effect exposure to cadmium has on AMH levels in premenopausal women. Our results suggest that cadmium exposure affects AMH concentration depending on the specific age group. When cadmium concentration is increased, the concentration of AMH is decreased in the age group of 30–35-year age group.

Very few previous epidemiologic studies have been conducted on the correlation between cadmium exposure and AMH concentrations in women. An earlier study, which examined the effect of cadmium exposure on AMH concentrations among 117 women, found that cadmium exposure had a positive association with AMH concentration in borderline statistically significant [15]. However, our findings differ from the findings of that earlier study because the characteristics of the subjects in both studies were very heterogeneous. There are many differences between the two studies, including the distribution of the subjects’ age, cadmium concentration, and AMH concentration. In the earlier study, the mean age of the subjects was 26.5 ± 5.6, the median AMH concentration was 11.71 (1.00–123.29), and the median cadmium concentration was 0.46 (0.17–2.98). However, in our study, the mean age of subjects was 36.22 ± 3.86, the median AMH concentration of 3.62 (1.88–6.01), and the median cadmium concentration was 0.96 (0.77–1.22). In particular, age has been known to be a crucial determinant of cadmium exposure and AMH concentration, respectively. As age increases, exposure to cadmium increases and the concentration of AMH decreases [1, 16]. Because the age range in the earlier study is about 10 years lower than the age range in our research; the cadmium concentrations were lower and AMH concentrations were higher in their study than in our research. In addition, given that the concentrations of cadmium and AMH vary with age, it is more reasonable to analyze the relationship between cadmium and AMH at similar ages after stratification. On the other hand, several studies have determined the effect of cadmium exposure on the reproductive system in men. A current review has outlined epidemiological observational findings for environmental and occupational exposure in humans through experimental studies in humans and animals [8]. These reports suggested that cadmium causes structural damage to testis vascular endothelium, which ultimately results in necrosis of the testis as well as affecting the blood-follicle-barrier (BFB) integrity, possibly leading to the development of autoimmunity against germ cells. Furthermore, cadmium might affect both spermatogenesis and testis by directly affecting inflammation mediators as well as pro-apoptotic and anti-apoptotic factors. In an in vitro study on pre-pubertal pig testis, Sertoli cell culture has shown that CdCl2 can adversely affect cell viability in a dose-dependent and time-dependent manner. The results showed that CdCl2 treatment can induce impaired function of the Sertoli cells as demonstrated by the reduction in INH-B and AMH secretion. CdCl2 also disrupts FSH receptor responsiveness (measured by a reduction in E2 production) and induces cell apoptosis. [17]. The molecular weight of AMH is 140 kDa. It is semi-restricted by normal functioning BFB. The disruption of the BFB could potentially cause an increase in the release of AMH from the follicles to circulation, thus theoretically explaining the increase in serum AMH in males [18]. The present study confirmed that cadmium exposure could affect AMH levels in women, similar to in men. The effect of cadmium exposure on the AMH concentration was also affected based on the age of the subjects. It is known that cadmium can alter the cycle pattern of oxidoreductase gene expression. Cadmium can also alter the clock gene in the neuroendocrine axis because it induces oxidative stress and regulates daily changes in HHG axis activity [19]. A separate review has outlined cadmium-induced neuroendocrine disruption of the hypothalamus-pituitary-gonad (HHG) axis [3]. Cadmium interferes with the regulatory mechanisms of this physiological axis by altering the neurotransmitters involved at the hypothalamic level, changing gonadotropin hormone secretion, and affecting testicular or ovarian structure and activity. These effects can be related to the periodic rhythm of the physiological axis associated with aging. Thus, there was also a study about cadmium toxicity, depending on age, in male rats. According to this study, the accumulation of cadmium in the hypothalamus, pituitary gland, and testis varied according to age. Cadmium accumulated in the three organs of young adult male rats but not in the hypothalamus and pituitary glands of older male rats. When they were exposed to cadmium, norepinephrine, dopamine and serotonin metabolism in the hypothalamus. Also levels of plasma LH and testosterone were different in young and old male rats. Thus, the study reported that the neurochemical levels of male rats and daily toxicity of cadmium in the reproductive axis were different in young and old rats. Since our study involved only women, it is difficult to interpret it with regard to previous studies. However, the results showed statistically different significance by age group. This suggests that there may be an association between AMH and cadmium only at a particular age, or there may be a difference in the mechanism depending on age, as a reference.

In this study, we surveyed the subject’s history of disease through questionnaires. Pelvic ultrasonography was used in order to determine any gynecologic diseases. In addition, environmental exposures were determined for subjects. These were the key strengths of this study. However, this study has limitations. First, it was a cross-sectional study that could not take time into consideration. In addition, all participants were healthy volunteers which might create bias research results. Although we have adjusted for the possible confounding effects of a number of covariates, there is always the possibility of remaining residual confounders, such as unmeasured grain intake in this study. It is also important to consider endocrine disruptors (ECDs), such as BPA and phthalates which are known to affect vital hormones, in examining the effects of heavy metals and hormones. Bias, such as co-exposure or collinearity, are well-known problems in epidemiological analysis. They occur because of a very high correlation due to common causes, such as shared cause, metabolic pathway, and other factors [20]. Therefore, the present study first identifies the single effects of cadmium and AMH; it then confirms the subsequent effects of the multi-pollutant approach (such as lasso, PCA, and factor analysis) on the combined effect of cadmium and ECDs on AMH [21].

## Conclusions

This study provides evidence suggesting that environmental exposure to cadmium may alter the level of AMH in premenopausal women, depending on their age.

## Abbreviations

AMH: Anti-Mullerian hormone
BMI: Body mass index
CAP: College of American Pathologists
CDC: By Centers for Disease Control and Prevention
LAMP: Lead and Multielement Proficiency
LOD: Limit of detection
PCOS: Polycystic ovary syndrome
TGF-β: Transforming growth factor-beta
